# Diet Management of Patients with Chronic Kidney Disease in Bariatric Surgery

**DOI:** 10.3390/nu15010165

**Published:** 2022-12-29

**Authors:** Marta Potrykus, Sylwia Czaja-Stolc, Sylwia Małgorzewicz, Monika Proczko-Stepaniak, Alicja Dębska-Ślizień

**Affiliations:** 1 Department of General, Endocrine and Transplant Surgery, Medical University of Gdansk, 80-211 Gdańsk, Poland; 2 Department of Clinical Nutrition, Medical University of Gdansk, 80-211 Gdańsk, Poland; 3 Department of Nephrology, Transplantology and Internal Medicine, Medical University of Gdansk, 80-952 Gdańsk, Poland

**Keywords:** obesity, bariatric surgery, chronic kidney disease, diet, nutritional management

## Abstract

Morbid obesity is considered a civilization disease of the 21st century. Not only does obesity increase mortality, but it is also the most important cause of the shortening life expectancy in the modern world. Obesity is associated with many metabolic abnormalities: dyslipidemia, hyperglycemia, cardiovascular diseases, and others. An increasing number of patients diagnosed with chronic kidney disease (CKD) are obese. Numerous additional disorders associated with impaired kidney function make it difficult to conduct slimming therapy and may also be associated with a greater number of complications than in people with normal kidney function. Currently available treatments for obesity include lifestyle modification, pharmacotherapy, and bariatric surgery (BS). There are no precise recommendations on how to reduce excess body weight in patients with CKD treated conservatively, undergoing chronic dialysis, or after kidney transplantation. The aim of this study was to analyze studies on the bariatric treatment of obesity in this group of people, as well as to compare the recommendations typical for bariatrics and CKD.

## 1. Introduction

Morbid obesity is now considered a civilization disease of the 21st century. A total of 1.9 billion adults, which corresponds to 39% of the world population, were estimated to be overweight globally in 2015 [1]. Obesity not only impairs physical and mental health but also has many economic consequences for national health care systems [2]. At an individual level, patients with obesity have an increased risk of major health problems such as hypertension, osteoarthritis, dyslipidemia, type 2 diabetes mellitus (DM2), coronary heart disease, stroke, gallbladder disease, sleep apnea, respiratory problems, and some cancers (endometrial, breast, and colon [3]. As a result, not only does obesity increase mortality, but it is also the most important reason for reduced life expectancy in the modern world. There are many metabolic abnormalities associated with obesity: dyslipidemia with increased triacylglycerol (TAG) and low-density lipoprotein (LDL) cholesterol concentrations and decreased high-density lipoprotein (HDL) cholesterol concentrations, dysregulation of glucose homeostasis with fasting and postprandial hyperglycemia and hyperinsulinemia, multiorgan insulin resistance, and liver steatosis are the hallmark features of obesity-related metabolic dysfunction also known as metabolic syndrome [4,5]. 

Little attention is paid to the fact that obesity increases the risk of developing chronic kidney disease (CKD), the incidence of which is 10.6% at stages 3–5. Studies show that CKD is more common than diabetes [6]. Obesity contributes to chronic oxidative stress, inflammation, and renal fibrosis, leading to progressive kidney damage and the need for renal replacement therapy (RRT) [7]. The exact mechanism of the influence of obesity and adipose tissue on kidney damage was presented in our previous publication [8].

Currently available treatments for obesity include lifestyle modifications, pharmacotherapy, and bariatric therapy. At each stage of obesity treatment, the patient should be encouraged to follow dietary recommendations and to exercise appropriately. Currently, pharmacotherapy (incretin family drugs or bupropion/naltrexone) is used more and more often. The latest therapeutic options indicate that, in some cases, drugs registered for the treatment of obesity may be helpful in combination with bariatric surgery [9].

Still, the most effective method of treating obesity is bariatric surgery (BS), which is also used in patients with co-morbid CKD. BS allows for the improvement of patients’ clinical conditions and reduces the severity of metabolic disorders. For patients with end-stage renal disease (ESRD), the weight loss achieved with BS often allows for a kidney transplant [10]. Patients should follow a proper diet before and after BS. Nutritional requirements are even more crucial for CKD patients. This study aims to review and summarize the literature on obesity and bariatric surgery among CKD patients as well as dietary recommendations for the previously mentioned group of patients. Articles on this topic were searched in the Pubmed and WorldWideScience databases for the following keywords “obesity”, “bariatric surgery”, “chronic kidney disease”, “diet”, “nutritional recommendations”, “nutritional management”, and their various combinations. The bibliography of selected publications was also analyzed. Publications in English and Polish were included.

## 2. Obesity and Chronic Kidney Disease

There is an increase in the incidence of CKD, which is usually diagnosed relatively late when nephroprotective actions bring little benefit. For this reason, the number of people requiring RRT, including dialysis and kidney transplantation, is also increasing [11]. CKD is divided into five stages according to the glomerular filtration rate (GFR). RRT is usually started when the GFR is lower than 15 mL/min/1.73 m^2^ or earlier when it is accompanied by, among others, malnutrition, overhydration, and severe uremia [12].

Overweight and obesity are factors contributing to the increased incidence of CKD. It is estimated that excessive body weight may contribute to up to 30% of CKD cases [13]. Obesity leads to functional and structural changes in the kidneys. Hyperfiltration and proteinuria, which are caused by glomerulosclerosis and tubulointerstitial fibrosis, are observed. These disorders are called obesity-related glomerulopathy (ORG) [14]. Excess body weight leads to chronic inflammation, oxidative stress, lipotoxicity, and impaired adipokine secretion, which also adversely affects kidney function [7].

Despite the existence of the so-called obesity paradox (reverse epidemiology), i.e., a positive correlation between excess body weight and the higher survival rate of patients undergoing dialysis, obesity may be an obstacle in qualifying for kidney transplantation [15]. Both donors and transplant recipients may be disqualified due to excess body weight. The body weight cut-offs that qualify for transplantation depend on the principles of the treatment center and the individual state of health of the patient. In most centers, body mass index (BMI) over 40 kg/m^2^ is a contraindication to the procedure, and over 35 kg/m^2^ is a relative contraindication [16]. The number of kidney transplant recipients (KTRs) with a BMI over 30 kg/m^2^ doubles every 15 years [17]. Obesity during transplantation is associated with poorer perioperative outcomes in terms of length of surgery and hospitalization, wound infections, delayed graft function, incisional hernia, and other complications [18,19].

According to data from 2013, over 25% of all living kidney donors are obese at the time of surgery [20]. Obesity increases the risk of ESRD among living kidney donors. Locke et al. observed that obese kidney donors had a 1.9-fold higher risk of post-donation ESRD when compared with non-obese donors. An increase in BMI of more than 27 kg/m^2^ before organ donation was associated with a 7% increase in ESRD risk after kidney donation [21].

## 3. Bariatric Surgery

Regarding the treatment of obesity, Capehorn et al. suggested a 4-tier framework to treat overweight and obesity systematically. The treatment starts with primary activity in tier 1—the general physician usually coordinates this phase. Tier 2 is a tighter regiment of lifestyle changes in preferably a group setting. The last conservative treatment step is tier 3—a step based on a multidisciplinary team with obesity specialists (mostly physiotherapists, psychologists, and nutritionists). The fourth tier is designed for individuals with a very severe and complex form of obesity, mostly for patients with BMI > 40 kg/m^2^ or BMI > 35 kg/m^2^ with obesity-related comorbidities and constitutes the surgical treatment of obesity [22].

These guidelines are still widely used. However, there is increasing evidence that bariatric procedures should also be considered for patients with DM2 and a BMI of 30 to 35 kg/m^2^ if hyperglycemia is inadequately controlled despite optimal medical treatment for DM2. Substantial evidence indicates that surgery results in greater improvements in weight loss and DM2 outcomes compared with nonsurgical interventions, regardless of the type of procedures used [23]. 

Bariatric procedures are divided into three types: restrictive, malabsorptive, and combination procedures (with a restrictive and malabsorptive component).

Restrictive surgical procedures lead to the decreased size of the stomach and reduced feeling of hunger or cause early satiety with smaller volumes of food [24,25]. Surgical procedures in this category include gastric banding (GB), vertical-banded gastroplasty (VBG), and sleeve gastrectomy (SG), where GB and VBG are methods, which are performed more and more rarely. SG is the most popular procedure, nowadays number one worldwide [26,27].

Malabsorptive procedures decrease the absorption of nutrients by excluding food from segments of the alimentary tract by either shortening the length of the tract or by bypassing anatomical segments or inter-transposing various segments of the bowel [28]. The first of these procedures were done in the fifties and the sixties, namely the jejunal–ileal bypass [29], the duodenojejunal bypass (DJB) [30], and later the biliopancreatic diversion (BPD) either with or without the duodenal switch [28,31].

The combination of both types of surgery aims to benefit from the restrictive and malabsorptive procedure. The Roux en-Y Gastric Bypass (RYGB) or One Anastomosis Gastric Bypass (OAGB) are the most well-known examples. While performing this procedure, a small pouch is created by performing a partial gastrectomy followed by creating an anastomosis of the small pouch to the jejunum (gastro-jejunostomy). Then, a bypass is achieved by identifying the transected stomach remnant and its attached segment of the duodenum and proximal jejunum, which is then mobilized at the jejunal end (the Roux en-Y limb), to be anastomosed to a distal segment of the jejunum to form a jejuno-jejunostomy [32].

The OAGB consists of a long conduit from below the crow’s foot extending up to the left of the angle of His. The stomach is divided, and a small, long tube is created, which becomes the pouch, similar in shape to the pouch of SG. OAGB has a 2–3 cm gastro-jejunal anastomosis to an anti-colic loop of jejunum 150–200 cm distal to the ligament of Treitz. The power of OAGB comes from the fact that it is both a “non-obstructive” restrictive procedure and has a significant fatty food intolerance component with minimal malabsorption [33].

According to the 2016 International Federation for the Surgery of Obesity and Metabolic Disorders (IFSO) survey data from 58/62 IFSO Societies, the total number of bariatric/metabolic procedures performed in 2016 was 685,874. The most common primary surgical bariatric/metabolic procedure was laparoscopic sleeve gastrectomy (LSG) (340,550; 53.6%), followed by LRYGB (191,326; 30.1%), and OAGB (30,563; 4.8%) [26,27].

BS results in durable excess weight loss (EWL). It reduces or at least improves comorbidities, improves the quality of life (QoL), and increases lifespan [34]. In addition to weight loss, BS impacts the resolution of DM2, metabolic syndrome, and cardiovascular diseases [35].

There is an increasing interest in metabolic improvements, which are observed even before any significant weight loss [36]. Walter Pories was the first to show this in 1992. He suggested that the beneficial effects of bariatric surgery go beyond weight loss [37]. It is known that BS can also improve the outcomes of patients suffering from many respiratory, cardiovascular, and metabolic diseases [38,39,40,41,42].

It is a well-known fact that some of the gut hormones modified by BS have anorectic actions and can significantly affect postoperative weight loss [43]. It is also recognized that macronutrient as well as micronutrient malabsorption is a complication of BS and it is not a desired mechanism of action for its long-term efficacy. Lower absorption of food and some metabolites leads to their deficiencies [44].

There is also a considerable number of publications describing the BS procedures among patients with a BMI lower than 35 kg/m^2^ and assessing the relationship between the gut hormone profiles and DM2 remission. The study by Celik et al. presents glycemic control. It was assessed in a prospective cohort of patients who had a Diverted Sleeve Gastrectomy with Ileal Transposition (DSIT), OAGB compared with SG in the first 30 days after BS. This study proved that the DSIT and OAGB were superior to SG in achieving glycemic control, defined as fasting glucose lower than 126 mg/dL [45]. These improvements in glycemic control after DSIT/OAGB are based on the Foregut–Hindgut hypothesis [46]. Transfer of the distal part of the ileum just below the pylorus that digested food stimulates producing hormones such as glucagon-like peptide 1 (GLP-1), peptide YY (PYY), and oxyntomodulin—hormones known for their anti-diabetogenic action. The increased blood levels of these hormones are found after other BS, such as RYGB, LSG, or BPD-DS. Despite the increasing interest in the metabolic aspect of BS, many issues of comorbidities resolution still need to be explained [47].

Surgical interference with the anatomy of the gastrointestinal tract can cause significant changes in the composition of the gut microbiome, which may affect the composition and number of various metabolites produced by intestinal bacteria. Research on this phenomenon has developed significantly over the past decade and suggests an association between microbiome changes after bariatric BS and patient health, especially in the context of metabolic disturbances [48,49].

## 4. Bariatric Surgery in Patients with Chronic Kidney Disease

BS is performed on CKD patients treated conservatively, on dialysis, before and after kidney transplantation. Weight loss in the above-mentioned groups of patients brings positive effects. Rapid and sustained weight loss due to BS enables patients with ESRD to stop dialysis treatment and receive a transplant. Additionally, pre-transplant metabolic surgery may reduce the risk of mortality and graft failure. On the other hand, it increases the pool of people who can donate an organ [19]. Weight loss slows disease progression in non-dialysis patients [50].

In 2022, a clinical practice guideline by the DESCARTES Working Group of ERA was published on the management of obesity in kidney transplant candidates and recipients. According to the guidelines, BS should be considered in kidney transplant candidates and recipients with a BMI ≥ 40 kg/m^2^ or a BMI ≥ 35 kg/m^2^ and at least one serious obesity-related condition that can be mitigated by weight loss. The suggested surgical method is LSG [9]. The 2020 Kidney Disease Improving Global Outcomes (KDIGO) recommendations suggest that obesity should not be a contraindication to kidney transplantation, but suggest weight loss interventions for obese candidates before transplantation [51].

Among the complications of DM2 is diabetic kidney disease (DKD). Its pathophysiology is associated with the metabolic disorders accompanying both DM2 and obesity, namely hyperglycemia, dyslipidemia, and arterial hypertension. DKD develops in approximately 40% of diabetic patients and significantly increases all-cause and cardiovascular mortality in this patient group [52]. Madsen et al. investigated the effect of RYGB on the remission of DM2 and its vascular complications. Due to the BS, 74% of the operated patients achieved remission of DM2 after one year after the surgery. Additionally, RYGB reduced the risk of vascular complications and reduced the development of DKD [53]. Bariatric procedures not only reduce the risk of developing DKD but also improve the results of patients suffering from it. Heneghan et al. reported that in obese patients with moderate and severe albuminuria, BS caused, in 58,3% of patients, remission of albuminuria at a 5-year follow-up [54]. Similar conclusions were drawn by Canney et al., who in their study achieved, at 13-months follow-up, diabetic albuminuria remission in 78% of patients undergoing RYGB [55].

Additionally, BS improves kidney function independently of improvements in glycemia. In a study where DM2 was not an inclusion criterion, metabolic surgery reduced the risk of eGFR decline by more than 30% by 58% and decreased serum creatinine by 57% [56]. In a long follow-up study, BS reduced the incidence of ESRD by over 70% approx. 18 years after surgery [57].

It appears that the improvement in the parameters of CKD is independent of postoperative weight loss, glycemic control, and blood pressure [52]. Table 1 shows the results of the studies of patients with CKD undergoing BS.

Studies show many benefits of BS among CKD patients. On the other hand, the results of the meta-analysis show that patients on chronic dialysis have a significantly higher risk of postoperative mortality and myocardial infarction after bariatric surgery compared to patients without renal failure [64]. However, it is known that obesity also increases cardiovascular risk, so it is necessary to compare the risks associated with BS and those of long-term obesity.

## 5. Diet before and after Bariatric Surgery

### 5.1. Patients without Chronic Kidney Disease

The diet before a bariatric procedure should aim to appropriate caloric content, the patient’s nutritional preferences, and economic possibilities. It should determine the form and duration of the patient’s physical activity and accompanying diseases. Although preoperative weight reduction reduces 30-day mortality and improves treatment outcomes, it should not be mandatory but recommended and supported by the therapeutic team. Compulsory weight loss and disqualification of patients by failing to meet this criterion results in a reduction in the number of patients qualified for surgery and an increase in obesity complications in the group of rejected patients [65].

A relatively small reduction in the amount of calorie intake (a daily deficit of 500–1000 kcal) is the most crucial component of the diet program. A deficiency of 7000 kcal of consumed food in relation to the body’s energy needs causes a loss of 1 kg of body weight. In order to lose about 0.5–1 kg body weight weekly, it is best to consume 600–1000 kcal less than the body needs every day. Recommendations regarding the specific caloric content of the diet should be selected individually to the health condition, body composition, and physical activity of the patient. Very restrictive diets such as low-calorie diets (LCD) or very low-calorie diets (VLCD) are increasingly being abandoned among CKD patients due to the risk of muscle loss [66]. On the other hand, there are reports on the beneficial effects of using a very low-calorie ketogenic diet (VLCKD). In the case of patients with CKD, it is necessary to consult a nephrologist before using the diet, because its use may lead to an exacerbation of the disease [67,68].

The daily requirement for nutrients during a reduction diet before bariatric surgery:

(1) Carbohydrates—45–50% of energy,
(2) Proteins—20–25% of energy, and
(3) Fat—20–25% of energy (saturated fatty acids <7%, cholesterol <200 mg/day).

As part of the recommended caloric value, the energy should come from vegetables, fruits, cereal products rich in fiber, and low-fat products among meat and dairy products. Diet should include mainly food products with a low GI, containing complex carbohydrates and a lot of fiber, and of fats—monounsaturated fatty acids, e.g., olive oil. Recommended reduction diets safe for health are: based on the assumptions of the Mediterranean diet, the DASH diet, a low-energy diet with low GI, and a low-energy, balanced diet (recommended by European Guidelines for Obesity Management in Adults) [69]. During the reduction diet, drinking 6–8 glasses/day of fluids is recommended. A low-energy diet should consist of 4–5 meals (3 main and 2 small) eaten regularly every 3 h. The first meal should be eaten within 1 h after waking up, and the last one—2–3 h before going to bed. Appropriate heat treatment of meals is also important: cooking, steaming, baking in casserole dishes in aluminum foil or baking bags, and stewing [70].

After the bariatric procedure, the general dietary recommendation should be the adaptation of the patient’s eating behavior to the surgical procedure and the general qualitative aspects of a healthy nutrient-dense diet. In the early period, the patient should be informed that: eating too much may lead to postoperative complications and worse weight loss effects; an adequate daily supply of protein is necessary to prevent malnutrition; high-calorie foods containing large amounts of simple sugars and fat (e.g., sweets) should be avoided; after LRYGB, Mini—Gastric Bypass (MGB)/Omega Loop Gastric Bypass (OLGB) and BPD procedures, this recommendation is also intended to prevent the occurrence of the dumping syndrome [71,72].

### 5.2. Patients with Chronic Kidney Disease

In the case of obese CKD patients or KTRs, bariatric procedures may be used before transplantation and as a method of treating excess body weight after transplantation. Obese patients with CKD require a careful metabolic evaluation and individualized modification of their eating habits. The preparation of a patient with CKD for BS should be supervised by a team of specialists. Preparations should start early enough and should include a thorough nutritional assessment, assessment of diet and eating habits, long-term weight history, defining the expectations of surgery, dietary recommendations for pre-, peri-, and postoperative nutrition as well as psychological support [65].

Nutritional management in KTRs is an important determinant of the outcome of transplantation in terms of both morbidity and mortality. A properly planned diet can be used to prevent and alleviate many complications associated with transplantation, although the exact need for nutrients has not been fully established yet. Many scientific studies provide guidance on nutritional management in the pre-transplant period and in the early and long-term post-transplant periods. A comprehensive approach in the pre-transplant period should include a change in the diet and lifestyle of the patient, aimed at facilitating the correction or reduction in malnutrition, obesity, osteodystrophy, and hypertension. Unfortunately, there are no specific dietary recommendations for patients undergoing bariatric surgery [73,74].

As recommended for all bariatric patients, a preoperative weight loss program should begin before surgery and include a low-calorie diet that will allow the patients to lose 10% of their body weight. Additionally, nutrient deficiencies must be identified and corrected during this time. Additionally, patients with CKD must adhere to the nutritional limits associated with kidney disease, which vary depending on the stage of the disease and the treatment used. As there are no specific recommendations for patients with CKD undergoing BS, it seems that the method of preparing patients for surgery should be based mainly on the patient’s nutritional status, treatment results/laboratory results, comorbidities, and patient preferences with the help of an experienced dietitian or nutritionist [75,76].

Laboratory tests should include markers useful in assessing nutritional status such as serum levels of total protein, albumin, and prealbumin. In addition, low cholesterol may be a marker of chronic inflammation or protein-energy deficit. Anthropometric measurements should be used, taking into account the total body weight as well as the differentiation between fat mass and lean mass, for a quick diagnosis of nutritional deterioration. When assessing the patient’s body weight, the possibility of edema should be taken into account. The best solution would be to use body composition assessment tools. The recommended method of body composition analysis is dual-energy X-ray absorptiometry (DXA), which is considered the gold standard. However, due to limited access to this method, bioelectrical impedance analysis (BIA) is most often used. However, in people with morbid obesity, the results of the BIA measurement may be inaccurate For the assessment of the diet, standard tools can be used, for example, the most commonly used 24 h dietary recall or a 3-day food diary [77,78,79].

Appropriate dietary management in bariatric patients eligible for kidney transplantation may be associated with some difficulties and depends largely on the nutritional status of the patient. Patients after bariatric procedures are exposed to deficiencies in proteins, vitamins B, vitamin D, iron, zinc, and calcium [70]. These deficiencies in dialysis patients may even worsen. This is especially true for protein and calcium, B vitamins, or iron [80].

#### 5.2.1. Energy Requirements

Typical daily caloric intake in the first week after surgery is 400 kcal/day and increases to 800 kcal/day within a month. A few months after the procedure, patients should consume 1200–1500 kcal/day [81]. For CKD patients, intake can be higher, especially in dialysis patients. The Look AHEAD study showed the benefits of using a reducing diet of 1200–1800 kcal in adult patients with DM2 and GFR < 60 mL/min/1.73 m^2^ and albumin/creatinine ratio (ACR) > 30 mg/g [82].

#### 5.2.2. Protein

The severity of the disease, the treatment received as well as the nutritional status of the patient affect the optimal amount of protein intake. Maintaining an adequate nitrogen balance is necessary to maintain proper nutritional status and body composition. Insufficient supply of external amino acids contributes to increased nitrogen catabolism, which deepens the negative nitrogen balance. In CKD patients requiring low-protein diets, essential amino acid requirements can be met with pharmaceutical mixtures of essential amino acids and ketoacids [83,84]. After BS, recommendations for protein consumption increase due to the increased demand resulting from the surgery and the difficulty in consuming the recommended amount of protein and its reduced absorption, which is a consequence of the procedure. Protein consumption of CKD patients after bariatric surgery under conservative treatment should increase moderately, taking into account the protein restrictions resulting from impaired renal function. Daily protein intake in this group of patients should be within 0.8–1 g/kg ideal body weight (IBW). For the same reasons, the increase in protein supply also applies to patients undergoing renal replacement therapy. Dialysis patients should intake ≥1.2 g of protein/kg IBW, and KTRs ≥ 1.1 g/kg IBW [78].

#### 5.2.3. Phosphate

Both hyper- and hypophosphatemia deteriorate health and increase the risk of death in patients with CKD. Although hyperphosphatemia is a more frequent phenomenon, deficiencies in this element also occur in this group of patients. For this reason, recommendations for phosphorus intake should be based on up-to-date laboratory test results. Long-term consequences of increased levels of phosphorus are cardiovascular calcification and secondary hyperparathyroidism, which contributes to metabolic bone disease. In turn, severe hypophosphatemia leads to impaired bone mineralization, rhabdomyolysis, hemolytic anemia, central nervous system dysfunction, and respiratory failure [85].

A common restriction in people with CKD is the daily phosphorus intake of 800–1000 mg [86]. However, protein-rich products are a good source of this element. Therefore, in people at increased risk or diagnosed with protein-energy wasting (PEW), the restriction can be mitigated if the maintenance of a low-phosphate diet takes place at the expense of protein and energy consumption. Additionally, the absorption of phosphorus in the digestive tract varies depending on its source. Absorption of phosphorus from animal products is higher than from plant products and amounts to approximately 70% and 50%, respectively. The highest absorption occurs in highly processed foods and it amounts to almost 100%. Additionally, in processed foods, phosphorus is added in the form of food additives, and its added content is not included in the total content of this element in the product [87,88]. The nutritional recommendations after BS do not focus on the phosphorus intake of the patients. It is a common element, and deficiencies in the general population are rare. In the rare case where patients have too low a serum phosphorus concentration, the consequences of metabolic surgery, such as a drastic reduction in food consumption, and, in the event of malabsorptive procedures the reduction in absorption, may aggravate phosphorus deficiency and worsen the patient’s condition. On the other hand, patients with too high phosphorus levels without the possibility of compensating for it through dialysis therapy may need Food for Special Medical Purposes (FSMP). They can help the patients meet the protein requirements that are increased after surgery, without increasing the supply of phosphorus and other nephralgic elements in CKD. Products dedicated to patients with CKD with increased protein content but reduced phosphorus, potassium, and sodium content may be helpful both for patients with RRT and for those treated conservatively in the perioperative period [66,89].

#### 5.2.4. Calcium

Both patients with CKD and patients after BS have an increased risk of osteoporosis and fractures. Sudden weight loss and altered postoperative absorption together with a reduced nutrient intake result in impaired bone metabolism. Therefore, after the surgery, the supply of calcium should be between 1200 and 1500 mg, depending on the type of surgery, from all sources. Due to impaired kidney function and, thus, a reduction in the conversion of inactive vitamin D into its active form, calcium absorption is reduced in patients with CKD. In patients with moderate to advanced CKD, it may be necessary to limit calcium intake to 800–1000 mg daily from all sources. However, reducing the absorption of fats, mainly after RYGB, causes an increase in fatty acids in the intestines, which preferentially binds to calcium and not to oxalates, as is the case under physiological conditions. The lower availability of calcium for binding to oxalates increases the concentration of soluble oxalates that can be absorbed into the bloodstream. Oxalates are filtered in the kidneys and excreted unchanged in the urine [52,90]. Excess urinary oxalate can lead to its deposition in the kidney. In turn, oxalate crystals are associated with renal inflammation, fibrosis, and faster progression of CKD [91]. It is crucial not only to consume sufficient calcium but also to avoid excessive consumption of oxalate to prevent the formation of calcium crystals in the kidneys.

In patients with CKD undergoing BS, both conditions should be taken into account and the daily calcium intake should be adjusted to achieve normal laboratory values of serum calcium [86].

#### 5.2.5. Potassium

As with the recommendations for phosphorus intake, potassium intake should be adjusted to the patient’s laboratory results. Both hypokalemia and hyperkalemia have serious health consequences. Abnormal high potassium levels in serum increase the risk of cardiac arrhythmia and sudden death [92]. Although hypokalemia is much less common in CKD patients, it has equally serious consequences. Impaired potassium excretion by malfunctioning kidneys leads to hyperkalemia. However, hypokalemia can occur as a result of the loss of this element through the gastrointestinal tract in the event of vomiting or diarrhea. In addition, the use of non-potassium-sparing diuretics and exposure to low-potassium dialysate during dialysis sessions may also significantly reduce serum potassium levels. Low potassium levels magnify the detrimental cardiovascular effects of increased sodium levels. Severe cases of hypokalemia can lead to paralysis and life-threatening cardiac arrhythmias [93].

In patients with CKD who underwent BS, special attention should be paid to the prevention of constipation. Constipation causes a prolonged stay of fecal content in the large intestine and, consequently, it may lead to increased potassium absorption and an increase in its concentration in the serum [94].

Managing and preventing hyperkalemia and hyperphosphatemia require a multidisciplinary approach that entails reducing foods rich in these nutrients, adjusting medications that cause their elevated serum levels, and adding medications that reduce them [95,96]. Selected products with high and low potassium content are presented in Table 2.

#### 5.2.6. Sodium

Regardless of the stage of the disease, patients with chronic kidney disease should consume 1.8–2.3 g of sodium per day. Reducing sodium is designed to reduce the risk of cardiovascular disease and help reduce thirst and thus maintain fluid restrictions [86].

#### 5.2.7. Magnesium

Magnesium participates in many metabolic processes as an enzyme cofactor. Its concentration is regulated by ingestion with food, exchange with bones, intestinal absorption and renal excretion. Its recommended daily intake depends on the age, sex and health of the patient. In adult men it is 420 and 320 mg/day in women. According to the scientific literature, there is an inverted correlation between magnesium and increased cardiovascular risk. In bariatric patients, both pre- and postoperative magnesium levels were associated with better glycemic control, and postoperatively with a higher rate of remission of type 2 diabetes within 1 year after surgery [99]. In patients with CKD, serum magnesium concentrations depend on eGFR and medication. Antacid medicines and laxatives can cause an increase in magnesium levels, and diuretics can decrease it. The method of treatment also affects the level of magnesium. Both dialysate concentration in hemodialysis patients and immunosuppressants in transplant patients significantly affect magnesium levels. Therefore, the daily requirement of magnesium should be adjusted to its serum level [78].

#### 5.2.8. Iron

The diet of obese patients, although it is high in calories, is deficient in terms of vitamins and minerals. For this reason, nutritional deficiencies, including iron deficiency, are common in this group of patients. People who undergo metabolic surgery are additionally subjected to various factors related to the procedure, which increase the risk of iron deficiency, for example, reduced consumption of iron-rich food products. Iron absorption is also reduced after surgery due to decreased acidity in the stomach caused by the reduction in acid secretion and the use of drugs to increase gastric pH. Additionally, in the case of malabsorptive procedures, the surface of nutrient absorption, including iron, is reduced. Iron is among the key building blocks of erythrocytes, and its deficiency may lead to the development of anemia. Symptoms of anemia include weakness, fatigue, dizziness, and, in more severe cases, angina pectoris, dyspnoea (shortness of breath) at rest, and even hemodynamic instability, which is a life-threatening condition. Additionally, in pregnant women, anemia may result in low birth weight of the baby, pre-delivery, and increased perinatal mortality for both mother and baby [100].

In CKD patients, anemia is also a common complication of compromising the quality of life and increasing morbidity and mortality [101]. Due to impaired kidney function, the production of erythropoietin by the kidneys is reduced. Additionally, impaired renal clearance and chronic inflammation associated with CKD increase the plasma levels of hepcidin, a hormone that regulates iron levels. Elevated hepcidin levels reduce iron absorption in the duodenum as well as iron availability for erythropoiesis and also cause endo- and exogenous erythropoietin resistance [102]. In patients after kidney transplantation, apart from the decrease in the function of the renal allograft, there is another factor that exposes patients to anemia, namely immunosuppressive drugs [103]. The effect of immunosuppressants on iron metabolism is not fully understood. The mammalian target of rapamycin inhibitors (mTORis) appears to promote iron deficiency by stimulating hepcidin expression [104].

In patients after BS, the iron content in their supplement preparations should be from 45 to 60 mg; in the case of treatment of deficiencies, the values may increase to 150–200 mg. If uncompensated deficits occur, despite the use of supplementation, an intravenous iron infusion may be necessary [80].

In the recommendations for patients with CKD, the dose of oral iron supplementation should be at least 200 mg per day. It is also noted that supplements should not be taken with meals rich in dietary fiber. Supplements should not be taken with coffee or tea due to the presence of caffeine and tannins, which also adversely affect the absorption of iron in the digestive tract. Iron absorption is improved by vitamin C; therefore, it is recommended to supply it at the level of 250 mg. Oral iron supplementation is often insufficient, and intravenous iron is needed. The intravenous route of iron supply can rapidly replenish iron stores, but carries a greater risk of allergies and infections. For this reason, it is worth trying to introduce iron supplementation in the form of chetal, which is more easily absorbed than in the traditional form [105]. In the event of failure of this form of supply the intravenous dose of iron used should be considered individually. Excessive iron intake may be a source of oxidative stress and increase the risk of infection [106,107].

#### 5.2.9. Folic Acid

Surgical treatment of obesity increases the risk of folic acid deficiency. It is essential for DNA and RNA synthesis, cell division, and protein synthesis. In the prenatal period, it is extremely important, and it corresponds to the proper development of the neural tube and thus the development of the fetus. Additionally, together with vitamins B_12_, B_6_, B_2_, and zinc, they participate in the regulation of the homocysteine cycle and thus, reduce the risk of cardiovascular disorders [108]. Absorption of folic acid after BS may be disturbed mainly by two factors: increasing the intestinal pH and bypassing the flow of food through the section where folic acid absorption occurs to the greatest extent, i.e., in the duodenum and the initial section of the jejunum [108,109].

It might seem that patients with CKD are at risk of folic acid deficiency due to the low consumption of vegetables—products rich in folic acid, which are also a good source of potassium. Nonetheless, its abnormal serum level is not common in this patient population. Patients with CKD should consume the amount of folic acid recommended for the general population and introduce supplementation only in the case of deficiency of this component [86]. For this reason, it seems that the recommended folate intake for patients with CKD after BS could be in line with that for patients after BS. Multivitamin preparations used after surgery as a nutrient supplement should contain 400 µg/day of folic acid. In pregnant women or those who are planning to conceive a child, this amount should be between 800 and 1000 µg. In the case of supplementing deficiencies, the amount may increase to 1 mg [75].

#### 5.2.10. Vitamin B_12_

According to the Kidney Disease Outcomes Quality Initiative (KDOQI), vitamin B_12_ supplementation is recommended only in case of its deficiency in CKD patients. Its administration, together with folate, to reduce the risk of cardiovascular disorders in the case of hyperhomocysteinemia is not recommended as there is no evidence of their effectiveness [86]. Insufficient oral intake and malabsorption are the two leading causes of B_12_ deficiency in bariatric patients. Vitamin B_12_ bound to protein is not digested. Free vitamin B_12_ is formed in the presence of stomach acids and pancreatic proteinases. Only after combining free cobalamin with the intrinsic factor, it is possible to absorb this complex in the distal ileum [110]. The excision of a portion of the stomach, i.e., the loss of a significant amount of intrinsic factor (IF)-producing parietal cells, reduces the IF-mediated absorption of cobalamin. In addition, the reduction in gastric acidity by decreased acid secretion caused by the loss of acid secreting cells or the use of drugs reducing the pH also impairs the absorption of this vitamin [111]. Clinical symptoms of vitamin B_12_ deficiency following surgical treatment of obesity can range from megaloblastic anemia and peripheral neuropathy to neuropsychiatric symptoms. The body’s storage of vitamin B_12_ may last for 3–5 years; therefore, its deficiency may not appear shortly after the surgery, but after a longer period of time [100]. A dose of 350–500 µg/day orally or 1 mg monthly intramuscularly or 3 mg every 3 months or 500 µg intranasally is recommended for bariatric patients [71].

#### 5.2.11. Vitamin D

Vitamin D deficiencies are common in the general population and are even more common in obese patients. Additionally, BS increases the risk of this deficiency. Vitamin D levels and BMI are negatively correlated with each other. Obesity may reduce the bioavailability of vitamin D by sequestering the vitamin through adipose tissue. Non-alcoholic fatty liver disease, which is very common in obese people by reducing hepatic 25-hydroxylase, also impairs the production of vitamin D [112]. After the procedure, a dose of at least 3000 international units per day is recommended to obtain a vitamin D result >30 ng/mL. For patients with severely impaired absorption of vitamin D, the initial oral dose should be 50,000 IU 3 times a week, and for vitamin D_2_ or vitamin D_3_ from 3000 to 6000 international units per day. It is believed that using the D3 form may be preferable, although both are acceptable [81].

CKD results in vitamin D metabolism reducing its concentration and thus leading to metabolic bone disease and other symptoms of deficiency. Depending on the severity of CKD and the presence and severity of secondary hyperparathyroidism, vitamin D supplementation is recommended [113]. In patients with normal vitamin D concentration who have parathyroid hormone above the normal range, supplementation with an active form of vitamin D should be introduced [78]. In the CKD population, there are no specific safe doses for vitamin D. It seems that the treatment of deficiency should be more aggressive in this patient group than in the general population. However, periodic measurements of serum calcium and phosphorus should be used to detect disturbances in their levels caused by vitamin D supplementation. Particular care should be taken for patients taking calcium-containing phosphate binders and vitamin D active analogues [86].

#### 5.2.12. Other Fat-Soluble Vitamins

Malabsorptive procedures, through the impaired absorption of fats, can reduce the absorption of fat-soluble vitamins. The occurrence of deficiencies in bariatric patients after these surgeries is quite common. Too low vitamin A concentration occurs in almost 70% of patients four years after the procedure. In turn, the deficiency of vitamins E and K is not common; therefore, screening their abnormal levels is recommended only in patients showing symptoms of deficiency [71]. According to Mechanick et al., routine multivitamins post-WLS should include 5000–10,000 IU of vitamin A (depending on the type of surgery), 15 mg of vitamin E, and from 90 to 120 µg of vitamin K [81].

The daily requirements of patients with CKD do not differ significantly from the general population; therefore, the daily intake of vitamins E and K should be consistent with the normal DRI for the general population. In patients with end-stage CKD on renal replacement therapy, the routine intake of vitamins A and E is discouraged due to their potential toxicity. In patients with CKD or after kidney transplantation taking anticoagulants, vitamin K supplementation is also not recommended, as these drugs block its activity [86]. In patients with impaired renal function, the toxic dose is lower than in patients with healthy kidneys. The increased vitamin A levels in CKD patients appear to be the result of impaired metabolism of retinol to retinoic acid, which is excreted via the kidneys, and an increase in retinol-binding protein. High serum vitamin A has an osteolytic effect, which may contribute to the development of metabolic bone disease [114]. It seems that supplementation of vitamin A should not exceed the daily DRI, which is 900 µg for men and 700 µg for women [78]. The combination of chronic kidney disease and obesity surgery may lead to a depletion of vitamin A supplies and the need for its supplementation, despite its potential toxicity. Particular attention should be paid to pregnant women as too much vitamin A may cause a teratogenic effect [115].

#### 5.2.13. Fluids

**Figure 1:**
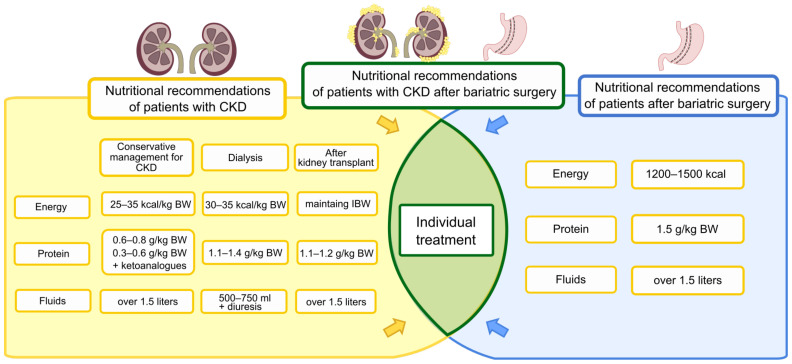
Nutritional recommendations of patients with CKD and after BS.

After BS, the oral supply will start with sugar-free clear liquids (preferably still water). On the third day after surgery, it is recommended to expand the diet slowly with other liquid products. The target recommended daily fluid intake is >1.5 L [116]. There are additional recommendations for fluid intake as described in the dumping syndrome section. Among patients with CKD with impaired urinary secretion, it is necessary to introduce fluid restrictions to reduce the risk of overhydration and edema. Such restrictions occur mainly among dialysis patients. The recommended fluid intake for maintenance hemodialysis patients is 500–750 mL/day in addition to daily urine output. Fluid restrictions should ensure that no more than 2–2.5 kg of weight gain between dialysis sessions is reached. It should be noted that fluid restrictions do not only apply to consumed beverages but also to dishes/products with a high water content, e.g., soups, yoghurts, fruits, and vegetables. Patients with CKD stages 1–4 and KTRs are usually advised to increase their fluid intake. Increased fluid intake is also recommended during the period of vomiting or diarrhea [117,118]. A summary of the nutritional recommendations of patients with CKD and after BS is shown in Figure 1.

#### 5.2.14. Dumping Syndrome

Dumping syndrome (DS) is caused by the too-rapid passage of high-osmotic chyme containing large amounts of easily digestible carbohydrates from the stomach to the small intestine. DS is divided into two types: early and late.

The symptoms of early postprandial syndrome begin within 10–30 min of eating a meal and last about 1 h. The rapid passage of hyperosmolar gastric contents into the small intestine leads to increased secretion of intestinal hormones and to a shift of fluids from the intravascular space into the intestinal lumen. This results in a decrease in plasma volume and the appearance of vasomotor symptoms such as hypotension, tachycardia, palpitations, hot flushes with drenching sweats, facial flushing, fatigue, and an urge to lie down after eating. Early dumping syndrome can lead to fainting. The shift of fluids into the intestine can cause the following symptoms: cramps, abdominal pain, bloating, nausea, and diarrhea.

The late postprandial syndrome occurs 1–3 h after a meal. The rapid passage of hyperosmotic gastric contents into the small intestine leads to the rapid absorption of glucose and to hyperglycemia. This leads to the stimulation of the secretion of the intestinal hormone—glucagon-like peptide-1 (GLP-1), which stimulates the excessive secretion of insulin and inhibits the secretion of glucagon, which leads to the occurrence of hypoglycemia [119,120,121].

In DS, in patients with or without CKD, the goals of nutritional intervention are to increase gastric emptying time, reduce food intake, and delay carbohydrate absorption. It is recommended to reduce the amount of food eaten. Patients should eat 5–6 meals a day with a small volume of about 150 mL (the volume of a cup). Meals should be eaten slowly, in a calm atmosphere. It is not recommended to drink during meals. Liquids should be consumed slowly, in small sips, 30 min before or 30–60 min after a meal. It is recommended to limit the consumption of easily digestible carbohydrates (glucose, fructose, glucose-fructose syrup, sucrose) and products with a high glycemic index. Each meal should consist of products that are a source of proteins, fats, and carbohydrates. Guar gum, pectin, and glucomannan supplementation may be considered to slow the rate of gastric emptying and glucose absorption [119,122].

## 6. Conclusions

Bariatric procedures are more and more often used in patients with obesity and simultaneously with CKD; the development of surgical methods allows for safer qualification of these patients for surgery. On the other hand, a comparison of dietary/nutritional recommendations for bariatrics and CKD, as well as consideration of the specific treatment of kidney diseases, indicate that the surgical management of patients with impaired renal function requires a multidisciplinary team of specialists. In addition to standard procedures before and after bariatric surgery, there should be cooperation with a nephrologist. It is also necessary to emphasize the role of an experienced dietician or nutritionist in the field of nutrition in nephrology.

It seems necessary to develop guidelines for the management of CKD patients after BS. They should take into account not only the principles of nutrition but also the possible supplementation of pro-, prebiotics, or other active substances that would have a beneficial effect on both weight loss and overall health improvement.

## Figures and Tables

**Table 1:** Literature review on the effect of BS on patients with CKD.

Reference	Health Condition	Sample Size	Exposure	Outcomes
CKD stages 1–5 (without dialysis)				
Navanethan et al. 2009 [58]	CKD stage 3	25	BS	GFR improvement at 6 and 12 months after surgery (from 47.9 to 61.6 mL/min/1.73 m^2^), a decrease in blood pressure was observed
Imam et al. 2017 [59]	CKD stages 3–4 group 1 group 2	714 714	SG/RYGB control	Bariatric surgery, especially the RYGB procedure, improves GFR for up to 3 years
Prasad et al. 2020 [60]	CKD stages 1–3 and 5	13	SG/RYGB	Significant decrease in protein and albumin excretion rate 6 months after surgery, and no significant changes in GFR
Kassam et al. 2020 [50]	ESRD without dialysis CKD stages 1–4	198 45	SG	The lasting effect on weight loss, reduction in comorbidities in both groups; improvement of renal function in patients with stage 3 CKD
		ESRD—dialysis		
Sheetz et al. 2020 [61]	group 1 ESRD and BS group 2 ESRD	1597 4750	group 1 BS	BS was associated with lower all-cause mortality and increased incidence of kidney transplantation
Kidney transplantation				
Cohen et al. 2019 [62]	KTRs CKD before KT	21 43	BS	BS before kidney transplantation was associated with a lower risk of graft failure than with BS after organ transplantation—disturbed tacrolimus levels among KTRs after BS
Schindel et al. 2019 [63]	KTRs BS group control group	30 50	SG/RYGB	Improvement of renal function, graft survival, and obesity-related comorbidities among kidney transplant recipients who underwent bariatric surgery

CKD—chronic kidney disease; BS—bariatric surgery; GFR—glomerular filtration rate; SG—sleeve gastrectomy; RYBG—Roux en-Y Gastric Bypass; ESRD—end-stage renal disease; KTRs—kidney transplant recipients.

**Table 2:** Selected products with high and low potassium content [97,98].

	Products	Potassium Content [mg of K per 100 g of Product]
Vegetables		
High potassium content	potatoes	420
	brussels sprouts	416
	broccoli	385
	green peas	353
	celery	320
Lower potassium content	onion	121
	cucumber	125
	butter lettuce	134
	Chinese cabbage	150
	green beans	211
Fruit		
High potassium content	avocado	600
	banana	395
	kiwi	290
	grapefruit	277
	apricots	275
Lower potassium content	blueberry	77
	cranberries	80
	pear	118
	watermelon	130
	strawberries	133
	apple	134
Nuts, seeds, dried fruit		
High potassium content	dried apricots	1666
	dried figs	938
	pumpkin seeds	810
	prunes	804
	cashew nuts	660
Lower potassium content	dried cranberries	49
	sesame	387
	pecans	360

## Data Availability

Not applicable.
